# Genomic Sequence of the Threespine Stickleback Iridovirus (TSIV) from Wild *Gasterosteus aculeatus* in Stormy Lake, Alaska

**DOI:** 10.3390/v16111663

**Published:** 2024-10-24

**Authors:** Alyssa M. Yoxsimer, Emma G. Offenberg, Austin Wolfgang Katzer, Michael A. Bell, Robert L. Massengill, David M. Kingsley

**Affiliations:** 1 Department of Developmental Biology, Stanford University School of Medicine, Stanford, CA 94305, USA; ambenj@stanford.edu (A.M.Y.); offenberg@stanford.edu (E.G.O.); awkatzer@stanford.edu (A.W.K.); 2 Department of Genetics, Stanford University School of Medicine, Stanford, CA 94305, USA; 3 Howard Hughes Medical Institute, Stanford University, Stanford, CA 94305, USA; 4 University of California Museum of Paleontology, Berkeley, CA 94720, USA; sticklemack@gmail.com; 5 State of Alaska Department of Fish and Game, Soldotna, AK 99669, USA; keeneye907@alaska.net

**Keywords:** megalocytivirus, iridovirus, threespine stickleback, *Gasterosteus*, B22

## Abstract

The threespine stickleback iridovirus (TSIV), a double-stranded DNA virus, was the first megalocytivirus detected in wild North American fishes. We report a second occurrence of TSIV in threespine stickleback (*Gasterosteus aculeatus*) from Stormy Lake, Alaska, and assemble a nearly complete genome of TSIV. The 115-kilobase TSIV genome contains 94 open reading frames (ORFs), with 91 that share homology with other known iridoviruses. We identify three ORFs that likely originate from recent lateral gene transfers from a eukaryotic host and one ORF with homology to B22 poxvirus proteins that likely originated from a lateral gene transfer between viruses. Phylogenetic analysis of 24 iridovirus core genes and pairwise sequence identity analysis support TSIV as a divergent sister taxon to other megalocytiviruses and a candidate for a novel species designation. Screening of stickleback collected from Stormy Lake before and after a 2012 rotenone treatment to eliminate invasive fish shows 100% positivity for TSIV in the two years before treatment (95% confidence interval: 89–100% prevalence) and 0% positivity for TSIV in 2024 after treatment (95% confidence interval: 0 to 3.7% prevalence), suggesting that the rotenone treatment and subsequent crash and reestablishment of the stickleback population is associated with loss of TSIV.

## 1. Introduction

Iridoviruses are a family of large, double-stranded DNA viruses. Their genomes are circularly permuted and terminally redundant, with unique regions ranging in length from 103 to 220 kbp [1,2]. Iridovirid genomes encode approximately 100 to 200 open reading frames (ORFs), which are present on both DNA strands and are primarily non-overlapping. Eaton et al. [3] identified a set of 26 core genes shared across the *Iridoviridae* family, with key catalytic, structural, and virulence-related functions [1]. Amino acid sequence comparisons and phylogenetic analysis using these core genes have been key criteria for assigning genus and species boundaries within the *Iridoviridae* family. 

The *Iridoviridae* family currently consists of two subfamilies and seven genera—*Alphairidovirinae* (Ranavirus, Lymphocystivirus, and Megalocytivirus), which infect poikilothermic vertebrates, and *Betairidovirinae* (Chloriridovirus, Daphniairidovirus, Decapodiridovirus, and Iridovirus), which infect invertebrate hosts. Megalocytiviruses are globally emerging aquatic pathogens with over 50 known freshwater and marine fish hosts [4]. Infections caused by megalocytiviruses were first reported in cultured red sea bream (*Pagrus major*) in Japan in 1990 [5]. Since then, megalocytivirus infections have caused numerous mass mortality events in food aquaculture and the ornamental fish trade [6,7,8,9,10]. While most outbreaks have occurred in East and Southeast Asia, megalocytivirus infections have also been reported in commercial fishes in North America, South America, Europe, and Australia [11,12,13,14,15,16,17].

The first known megalocytivirus infection of a wild North American fish was identified in threespine stickleback (*Gasterosteus aculeatus*) collected from coastal British Columbia, Canada, in 2007 and termed the threespine stickleback iridovirus (TSIV) [18,19]. Stickleback are non-commercial, small, bony fishes distributed widely throughout the northern hemisphere. Their repeated dispersal from oceanic habitats to freshwater lakes and streams, and rapid evolution of diverse morphological, physiological, and behavioral phenotypes, have made them a powerful model system for studying vertebrate evolution and adaptation [20,21]. Although well-developed genetics and genomics in the stickleback system provide a potential opportunity to investigate host–pathogen coevolution in natural environments, relatively few stickleback viruses have been identified and characterized [18,19,22,23,24,25].

In contrast to the high mortality rates of some megalocytivirus-infected fish reported in aquaculture, TSIV infection of the coastal Canadian stickleback was associated with relatively low-level mortality while fish were held in captivity, and seemingly healthy fish also contained viral lesions [18]. TSIV-infected stickleback also displayed cytomegalic cells with abundant amphophilic intracytoplasmic inclusions in many tissues, coagulative hepatocellular necrosis, and co-infections with other parasites.

Phylogenetic characterization based on the major capsid protein (*MCP*) and *ATPase* genes placed TSIV as a divergent sister taxon to the other megalocytiviruses that had been reported [18]. These other megalocytiviruses have been broadly called infectious kidney and spleen necrosis virus (ISKNV) but cluster into three genotypes: Genotype 1, represented by red sea bream iridovirus (RSIV); Genotype 2, represented by ISKNV; and Genotype 3, represented by turbot reddish body iridovirus (TRBIV) [26,27,28,29,30]. Since the discovery of TSIV, two other divergent megalocytiviruses have been characterized: scale drop disease virus (SDDV) in barramundi (*Lates calcarifer*) [31] and the European chub iridovirus (ECIV) in *Squalius cephalus* [32]. Although Waltzek et al. [18] suggested the partially characterized TSIV was divergent enough that it might merit a separate species designation, the International Committee on Taxonomy of Viruses (ICTV) currently recognizes two *Megalocytivirus* species: *Megalocytivirus pagrus1*, which includes the ISKNV, RSIV, and TRBIV isolates; and *Megalocytivirus lates1*, which includes the SDDV isolates. Additional information from the full genome sequence of TSIV will be a valuable resource to clarify the species designation of TSIV, elucidate genome evolution within the genus *Megalocytivirus*, and investigate virus–host coevolution in stickleback. 

Here, we assemble a nearly complete TSIV genome from an additional occurrence of TSIV found in a wild North American stickleback population in Stormy Lake, Alaska. Using this genome, we generate a core gene phylogeny to reassess the relationship between TSIV and other megalocytiviruses, and identify TSIV ORFs likely originating from more recent gene transfers from fish hosts and other viruses. We also determine the prevalence of TSIV in Stormy Lake before and after chemical treatment of the lake to eradicate invasive fish, and the prevalence of the virus following release of wild Stormy Lake stickleback into a nearby lake in Alaska.

## 2. Materials and Methods

### 2.1. Screen for Iridovirus-Infected Stickleback

Whole-genome sequence (WGS) data from 206 globally distributed stickleback were previously generated and mapped to the stickleback reference genome version gasAcu1-4 as described by Roberts Kingman et al. [33] (SRA accession PRJNA247503). To search for new examples of TSIV-infected stickleback, we extracted gasAcu1-4-unaligned reads using Samtools v. 1.13 [34] and searched for perfect matches to 27 bp kmers generated from the three partially sequenced TSIV genes previously reported in Vancouver Canadian fish [18]—the *MCP*, *ATPase*, and *DNA polymerase* (Genbank accessions HQ857785, HQ857786, HQ857784)—using bbduk.sh from BBtools v. 39.01 (sourceforge.net/projects/bbmap/).

### 2.2. Genome Assembly and Annotation

A WGS library was prepared with pectoral and caudal fin DNA from a single Stormy Lake 2012 stickleback (STMY_2012_42) using the NEBNext^®^ Ultra™ II DNA Library Prep Kit according to the manufacturer’s recommendations. The library was sequenced on two flow cells of the Illumina MiSeq platform using a 2 × 300 paired-end configuration. Raw reads were trimmed using Trim Galore v. 0.6.10 with a minimum quality of 20 and minimum length of 200 bp (https://www.bioinformatics.babraham.ac.uk/projects/trim_galore/; accessed on 8 February 2023). Trimmed reads were mapped to the stickleback reference genome version gasAcu1-4 using bwa mem v. 0.7.18 and unaligned reads were extracted using Samtools v 1.20 [34,35]. Due to high viral coverage, unaligned reads were subsampled to 0.5% using Seqtk v. 1.4 (https://github.com/lh3/seqtk; accessed on 17 August 2023) and de novo assembled using SPADES v. 3.15.2 [36]. Putative ORFs were predicted using GeneMarkS v. 4.3 with the virus setting [37]. To identify putative ORF function and homologs, we performed a BLASTP search of ORF protein sequences against the non-redundant protein sequences database (nr) and individual databases generated from 70 iridovirus genomes (Table S1) [38]. To confirm contig quality, reads were remapped to the STMY_2012_42 assembled contigs using bwa mem v. 0.7.18 and read depth was calculated using Samtools coverage with a minimum MAPQ = 3 (Samtools v. 1.20) [34,35].

Contigs that (1) contained at least one ORF with a homolog to a known iridovirus and (2) showed consistently high coverage levels (~53,000×) were included in the STMY_2012_42 isolate TSIV assembly (Genbank accession numbers PQ335173 and PQ335174). ORFs were classified into five categories based on shared protein sequence homology with (1) the 26 iridovirus conserved genes established by Eaton et al. [3], (2) other shared iridovirus ORFs, (3) ORFs typically found in poxviruses, (4) eukaryotic genes, or (5) no known proteins. Conserved domains were annotated using the NCBI Batch CD-search tool (https://www.ncbi.nlm.nih.gov/Structure/bwrpsb/bwrpsb.cgi; accessed on 5 September 2024). GC content was calculated using seqkit v. 2.3.1 [39]. Proksee (https://proksee.ca/; accessed on 7 September 2024) was used to visualize genome annotations and generate tracks for GC content and GC skew. To visualize structural organization and repeats within the TSIV genome, a dot plot was generated using the YASS web server with default parameters [40].

### 2.3. Phylogenetic Analysis

Iridovirus core genes established by Eaton et al. [3] were identified in the TSIV genome and 70 additional iridovirus genomes using BLASTP. Amino acid multiple sequence alignments were generated for each of 24 core genes using MAFFT v. 7.490 with default settings implemented in Geneious Prime v. 2024.0.3 [41]; transcription elongation factor TFIIS and deoxynucleoside kinase were excluded because they were not present in the TSIV and ECIV genomes, respectively. All 24 core protein alignments were concatenated in Geneious and maximum likelihood analysis was conducted using IQ-TREE v. 2.3.6 with default parameters and 100 non-parametric bootstraps to produce a phylogenetic tree [42,43]. Gaps in alignments were treated as unknown characters, representing no information [42]. Phylogenetic trees were visualized using TreeViewer [44]. The same alignment, maximum likelihood, and visualization methods were performed for TSIV ORF65 (poxvirus B22 protein) and the B22 genes from two other iridoviruses, three herpesviruses, and twelve poxviruses to produce a B22 phylogenetic tree (Table S2).

To compare the sequence similarity of the Stormy Lake TSIV isolate to the prior TSIV isolate [18] and other iridoviruses, we prepared pairwise identity matrices from the *MCP* nucleotide sequences and the concatenated 24 core gene amino acid sequences using the Sequence Demarcation Tool v. 1.0 with MAFFT v. 7.526 [41,45].

### 2.4. Collinearity Analysis

Synteny between TSIV and other megalocytiviruses was visualized using the gggenomes v. 1.0.1 package in R 4.2.3 [46]. Lines were drawn between genomes connecting the homologous iridovirus core genes and the *B22* gene. Genomes were arranged such that the first gene was the transmembrane amino acid transporter gene (ISKNV ORF1L) in the reverse orientation. To determine the relative orientation of the two TSIV assembled contigs, paired reads from STMY_2012_42 and STMY_X_2011_03 discordantly mapped to both contig1 and contig2 were extracted using Samtools v. 1.20 [34]. The positions of the forward and reverse paired reads on contig1 and contig2 were visualized using the tidyverse v. 2.0.0 package in R 4.3.3 to identify paired reads spanning contig ends [47]. 

### 2.5. Detection of TSIV in Stormy Lake and Wik Lake

Stickleback were captured from Stormy Lake, Alaska (60°46′51″ N 151°03′10″ W), in 2011, 2012, and 2024, and from Wik Lake (60°43′04″ N 151°15′03″ W) in 2024 using minnow traps. Stormy Lake 2012 stickleback were collected prior to rotenone treatment of the lake [48]. Fish were sacrificed in MS-222 using protocols approved by the Institutional Animal Care and Use Committee of Stanford University (protocol 13834), and whole fish were preserved in 70% ethanol. For Stormy Lake 2012, ethanol was removed and fish were stored at −20 °C. Similar positive rates were seen in the 2011 and 2012 cohorts after storage in liquid ethanol or by freezing, so we think it unlikely that the different storage methods determined the positive rates observed. DNA was extracted from pectoral fins for Stormy Lake 2011 fish (no caudal fin available) and caudal fins from all other fish, a known site of TSIV infection [18]. Fin clips were incubated at 55 °C overnight in 600 µL lysis buffer (10 mM Tris, pH 8, 100 mM NaCl, 10 mM EDTA, 0.5% SDS, Proteinase K (333 μg/mL), then mixed with 600 uL of phenol:cholorform:isoamyl alcohol 25:24:1 and centrifuged for 10 min at 20,800× *g*. The aqueous portion was mixed with 600 µL of chloroform and centrifuged for 5 min at 5000× *g*. DNA was precipitated by mixing the aqueous portion with 1 mL of 100% ethanol and 30 µL 3 M NaOAc (pH 5.2), and incubating 1 h to overnight at −20 °C. The sample was centrifuged for 15 min at 20,800× *g* at 4 °C and the resulting pellet was rinsed with 70% ethanol, then air dried and resuspended in 50 µL of TE, low EDTA buffer. Stormy Lake 2011 DNA samples were additionally purified using the OneStep PCR Inhibitor Removal Kit (Zymo, Irvine, CA, USA) according to the manufacturer’s protocol.

To screen fish for TSIV infection, PCR primers were designed to target a 166 bp sequence from TSIV *DNA polymerase* (Forward: 5′-GCCTTTTCGATGAGCTTGCG-3′, Reverse: 5′-GCCTCTCGGACGTAGACATG-3′) and a 600 bp sequence from the stickleback *Htra1a* gene as a control locus (Forward: 5′-TGCTCCTTTACTGTGTGTGCA-3′, Reverse: 5′-GAGACCAGGGGAGTTTGTGG-3′). PCR reactions were prepared in 10 µL volumes using 1× DreamTaq Green PCR MasterMix (ThermoScientific, Waltham, MA, USA), 0.5 µM of each primer, and 5 ng of DNA and amplified with the following protocol: (1) 1 cycle at 95 °C for 1.5 min, (2) 35 cycles at 95 °C for 30 s, 56 °C for 30 s, and 72 °C for 1 min, and (3) 1 cycle at 72 °C for 5 min. Amplicons were visualized by electrophoresis on a 2% agarose gel, and sequencing confirmed that the 166 bp and 600 bp amplicons corresponded to the TSIV *DNA polymerase* and control stickleback *Htra1a* locus, respectively. Viral positivity was calculated as the percentage of samples positive for the TSIV amplicons, and the 95% confidence interval of TSIV prevalence was calculated by the Wilson/Brown method [49].

## 3. Results

### 3.1. Screen for Iridovirus-Infected Stickleback

We searched 206 previously sequenced, geographically diverse, wild stickleback genomes for the presence of TSIV kmers that matched sequences from the three partial ORFs characterized in the 2007 Vancouver TSIV outbreak [18]. Of 156 Pacific North American, 49 Atlantic and European, and 1 Japanese fish, we only detected kmers for all 3 previously known TSIV genes in 1 fish collected from Stormy Lake, Alaska in 2011 (STMY_X_2011_03). Stormy Lake is a 6958 acre-foot postglacial lake located above the 60-degree latitude that has previously been studied for the presence of native and non-native fish and plant species [48] (see additional information in Section 3.5). Of 1,411,418 unmapped reads, 305 reads contained kmers from the *MCP*, 166 reads contained kmers from the *ATPase*, and 135 reads contained kmers from the *DNA polymerase*.

### 3.2. TSIV Genome Assembly and Annotations

**Figure 1:**
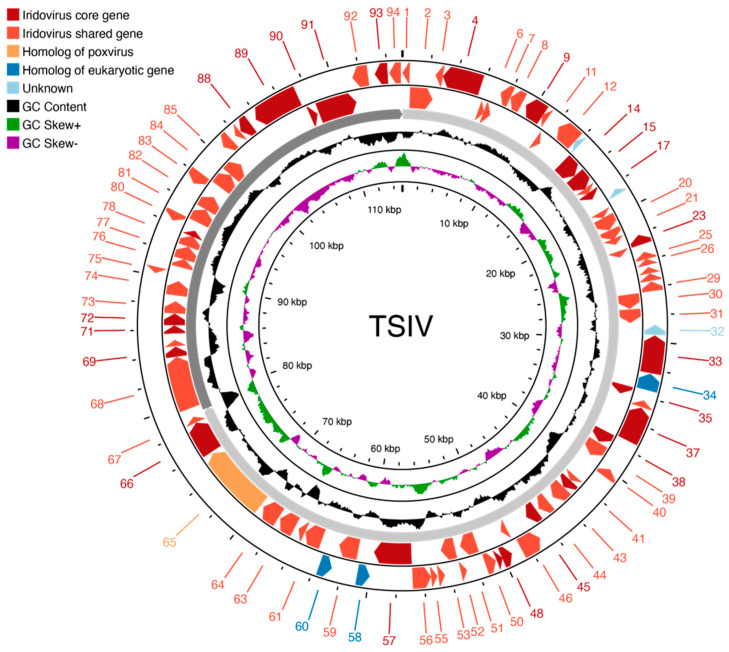
Map of the TSIV genome. The two outer circles represent predicted ORFs colored based on shared homology with proteins from other iridoviruses, poxviruses, or eukaryotic organisms. The next circle represents the breakpoints of the two contigs comprising the assembly. The two inner circles depict GC content and GC skew.

To assemble a high quality TSIV genome, we surveyed additional Stormy Lake fish for possible iridovirus infection (Section 3.5), and sequenced a TSIV *DNA polymerase* positive stickleback collected from Stormy Lake in 2012 (STMY_2012_42). Of 30,210,814 raw read pairs, 10,847,627 (35.9%) did not map to the stickleback reference genome. The subsampled reads produced an assembly of 128 contigs. Of these, only the two longest contigs contained predicted ORFs with homology to known iridovirus proteins and had consistently high read depth (~53,000×) compared to stickleback reference genome-mapped reads (~15×). We considered these two contigs to be the Stormy Lake isolate TSIV genome, which constitutes a length of 115,128 bp with 60.2% GC content and 94 putative ORFs (Figure 1, Table S3). The existence of two contigs rather than a single large contig suggests some TSIV sequence is still missing or hard to assemble. However, we did detect multiple sequence reads that spanned the ends of both contigs, indicating the likely orientation of the two contigs in the overall TSIV genome, and suggesting that unassembled gaps between contigs are likely small (Figure S1 and also see Section 3.3 below). The TSIV genome contains a minimal number of repetitive sequences, with a few direct repeats each shorter than 1.5 kb (Figure S2). 

Of the 94 predicted TSIV ORFs, only four ORFs showed no homology to any other proteins in the nr sequence database, although one (ORF80) had homology to hypothetical proteins in iridovirus genome-specific BLASTP databases. The remaining 90 ORFs share homology with other megalocytiviruses and include 25 of the 26 iridovirus core proteins described by Eaton et al. [3]. We did not detect the transcription elongation factor TFIIS in the TSIV genome (core gene 3N), which could reflect a true absence in the TSIV genome or an incomplete assembly. Although these 91 ORFs share homology with iridoviruses, the highest scoring BLASTP hits for 3 ORFs—ORF34, ORF58, and ORF60—are to proteins encoded in fish genomes—*Oryzias melastigma*, *Larimichthys crocea*, and *Melanotaenia boesemani*, respectively. While the ORF58 and ORF60 homologs lack functional annotation, ORF34 is predicted to encode TNF receptor-associated factor 2 (TRAF2). The TSIV TRAF2 is 398 aa, which is 18% shorter than the *O. melastigma* TRAF2 (485 aa) but contains both the N-terminal RING domain and C-terminal TRAF domain. The higher sequence identities of these three ORFs with fish than known viral proteins suggest they may have been acquired by more recent gene exchanges between iridoviruses and their hosts. 

The largest gene in the TSIV genome encodes a 1902 aa predicted transmembrane protein (ORF65). Although the top BLASTP hits for ORF65 are for related proteins in ECIV and SDDV, the majority of BLASTP hits are to B22 proteins in poxviruses. B22 proteins are typically found in chordopoxvirus genomes and have been determined to inhibit antigen presentation to T-lymphocytes and natural killer cells during infections, increasing poxvirus virulence [50,51,52].

### 3.3. Relationship of TSIV to Other Megalocytiviruses

**Figure 2:**
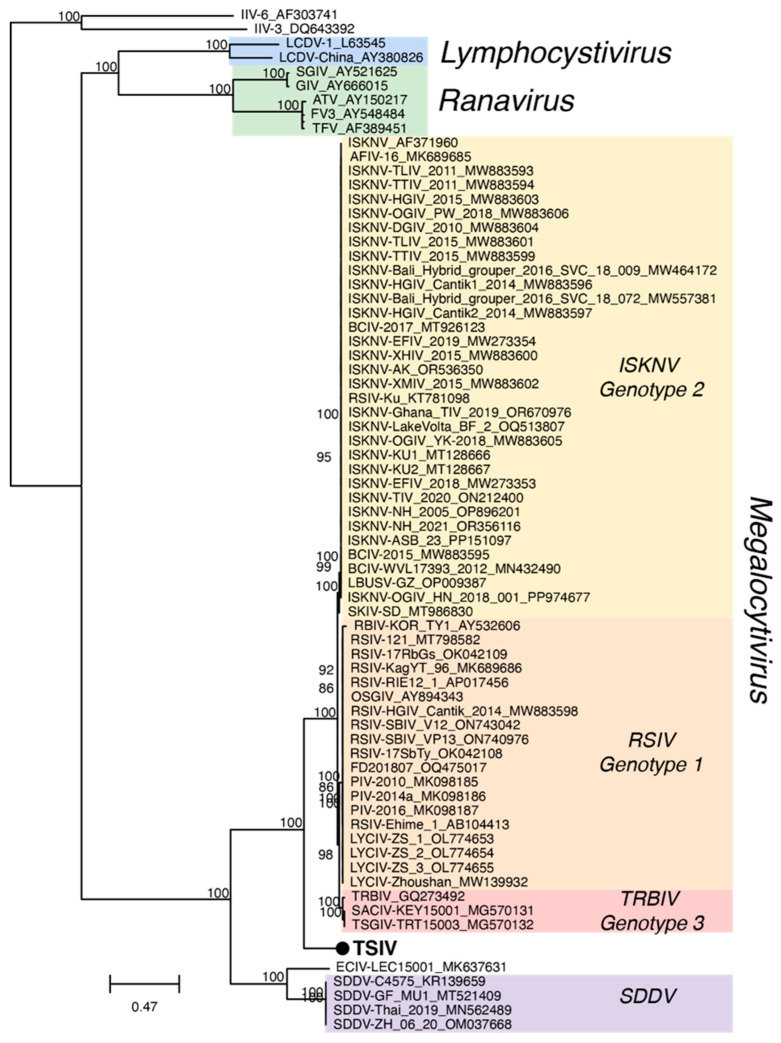
Phylogenetic tree of 71 iridoviruses based on the concatenated amino acid sequences of 24 core proteins. Bootstrap values ≥85 are labeled above each node. Branch lengths are based on the number of inferred substitutions, indicated by the scale bar. See Table S1 for additional information about each viral sequence.

Multiple sequence alignments of 24 iridovirus core proteins from TSIV and 70 other iridoviruses generated a concatenated alignment length of 18,140 amino acids. Maximum likelihood analysis of the resulting alignment produced a phylogenetic tree with well-supported nodes between established species but did not successfully resolve relationships within previously described ISKNV genotypes, which share very high sequence identity (Figure 2, Table S1). Consistent with prior analysis based on the MCP and ATPase, we observed strong support for TSIV as a sister taxon to the ISKNV isolates and distinct from the clade formed by SDDV isolates and ECIV [18,31,32]. Pairwise nucleotide identities of the *MCP* gene showed 99.5% shared sequence identity with the previously reported TSIV isolate [18], 79.1–80.5% identity with ISKNV isolates, 61.1–63.1% identity with SDDV isolates and ECIV, and 48.5–57.4% identity with non-megalocytivirus iridoviruses (Table S4). Pairwise amino acid identities of 24 concatenated core genes showed that TSIV shares 70.1–70.9% identity with ISKNV isolates, 49.2–49.5% identity with SDDV isolates and ECIV, and 29.2–36.7% identity with non-megalocytivirus iridoviruses (Table S5). In contrast, different members of the previously recognized ISKNV species group share 93.8–100% amino acid identity with each other, and different members of the previously recognized SDDV group share 99.9–100% sequence identity.

**Figure 3:**
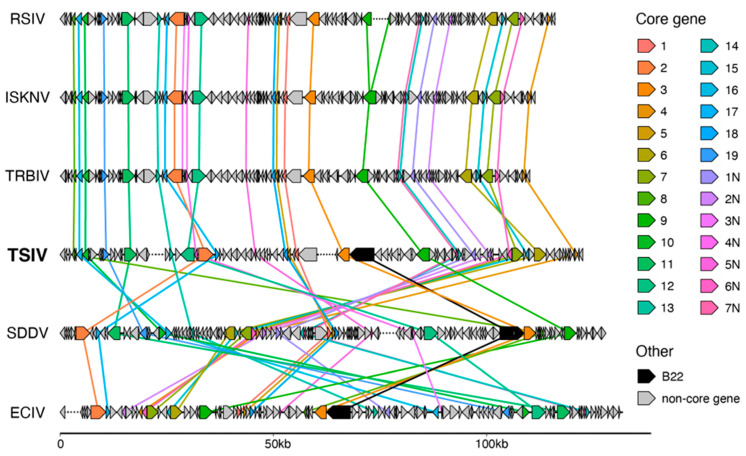
Comparison of collinearity between megalocytivirus genomes. Colors refer to the core genes as numbered by Eaton et al. [3]. Lines are drawn between homologous core genes and the *B22* genes in each genome. Dashed lines within genome tracks indicate positions where assemblies were rearranged and reconnected so that the transmembrane amino acid transporter gene is the first gene shown and is in the reverse orientation. See Table S1 for accessions used for each genome.

To compare overall genome organization, we visualized the relative positions of homologous iridovirus core genes in TSIV and other representative megalocytivirus genomes. The positions of reads discordantly mapped to both TSIV contig1 and contig2 suggested that the TSIV genome should be arranged such that the contig1 end is near the contig2 beginning and the contig2 end is near the contig1 beginning (Figure S1). Based on this arrangement, collinearity analysis shows that TSIV shares a similar genome organization to the ISKNV species group except for two inversion regions of approximately 10 kb and 16 kb (Figure 3). SDDV and ECIV are largely non-collinear with any of the other genomes represented.

### 3.4. B22 Phylogeny

**Figure 4:**
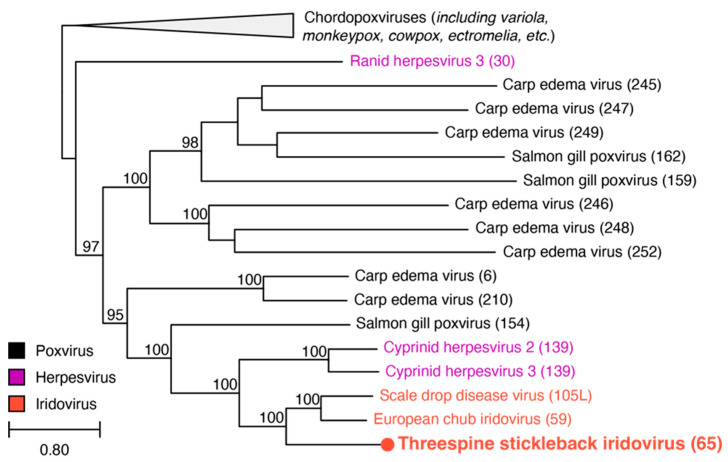
Phylogenetic tree based on the B22 amino acid sequences from iridoviruses, herpesviruses, and chordopoxviruses. ORFs from each viral sequence are indicated in parentheses. Bootstrap values ≥85 are labeled above each node. Branch lengths are based on the number of inferred substitutions, indicated by the scale bar. See Table S2 for additional information for each viral sequence.

To evaluate the relationship of the B22 protein encoded in the TSIV genome to other viruses, we also conducted a maximum likelihood analysis of the TSIV ORF65 amino acid sequence and B22 sequences detected in other iridoviruses (SDDV and ECIV), herpesviruses (CyHV3, CyHV2, and RaHV3), fish poxviruses (SGPV and CEV), and a representative selection of other chordopoxviruses (Table S2). The phylogenetic analysis supports at least two separate transfers of B22 from poxviruses to other viral lineages: (1) ORF30 in ranid herpesvirus 3 and (2) the clade containing the cyprinid herpesviruses and the megalocytiviruses (Figure 4). However, given the distant evolutionary relationship between herpesviruses and iridoviruses, the B22 ORFs in the cyprinid herpesviruses and megalocytiviruses likely stem from two different gene transfer events from poxviruses or potentially from the subsequent transfer of the B22 ORFs between a herpesvirus and a megalocytivirus. The absence of any B22 ORFs in ISKNV isolates but presence of B22 in the genomes of SDDV, ECIV, and TSIV, which occupy the basal positions of the megalocytivirus clade, suggest B22 may have been gained in the common ancestor of megalocytiviruses and subsequently lost in the ISKNV species. We note that B22 is located next to the same neighboring gene in the TSIV, ECIV, and SDDV genomes (core gene 3, encoding a putative NTPase I), further supporting a single ancestral acquisition of B22, followed by loss in the ISKNV group (Figure 3).

### 3.5. Detection of TSIV in Stormy Lake and Wik Lake

**Figure 5:**
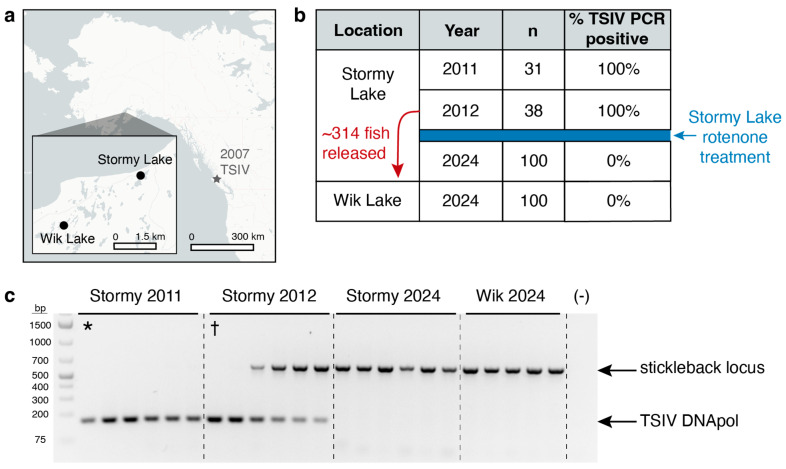
TSIV detections in stickleback from Stormy and Wik Lake. (**a**) Map depicting locations of Stormy and Wik Lakes in Alaska and 2007 TSIV outbreak described by Waltzek et al. [18]. (**b**) Location, year, and number of stickleback screened from Stormy and Wik Lakes and TSIV positive percentage by PCR. (**c**) Representative samples from PCR screen for TSIV presence. Each well represents an individual fish, including (*) the STMY_2011_X_03 individual detected by the kmer screen of the Roberts Kingman et al. dataset [33], and (†) the STMY_2012_42 individual used for TSIV genome assembly. The (-) lane shows the absence of amplified products from a water negative control.

Because we detected TSIV infection in the single Stormy Lake individual included in the Roberts Kingman et al. dataset [33], we sought to determine the prevalence of TSIV infection in other fish collected from this Alaskan lake. Stormy Lake is a freshwater lake located on the Kenai Peninsula of Southcentral Alaska. It drains into the Swanson River through a 1200 m outlet stream (Figure 5a). Due to the presence of invasive northern pike (*Esox lucius*) in Stormy Lake, fish passage between Stormy Lake and the Swanson River had been blocked by a fyke net since 2001. In an attempt to eradicate the northern pike, the Alaska Department of Fish and Game treated Stormy Lake with rotenone, a naturally occurring piscicide, in September 2012 [48]. Prior to rotenone treatment, an estimated 314 stickleback, along with other native fishes, were moved to net pens in nearby Wik Lake with the intent to return them after detoxification of Stormy Lake (Figure 5a). However, the mesh size of the pens was too large to contain the stickleback, which escaped into Wik Lake and consequently were not returned to Stormy Lake. Stickleback were observed in Stormy Lake in July 2014, their reappearance likely explained by recolonization from the Swanson River through the outlet stream after the fyke net was removed [48].

We developed a PCR assay to detect the presence of TSIV *DNA polymerase* in fin clip samples prepared from individuals collected in different years before and after the rotenone campaign. Sequencing of the PCR product amplified from Stormy Lake fish confirmed that the 166 bp product shows the expected sequence of TSIV *DNA polymerase*. All 31 of 31 stickleback collected from Stormy Lake in 2011, and all 38 of 38 fish tested from Stormy Lake in 2012 (collected in August, prior to rotenone treatment in September), were positive for the TSIV *DNA polymerase*, suggesting very high TSIV prevalence was maintained in two consecutive years in the wild Stormy Lake population (Figure 5b, 95% confidence intervals: 2011, 89–100% prevalence; 2012, 91–100% prevalence). We note that the amplicon band intensity varied in different individuals, and as the TSIV *DNA polymerase* amplicon increased in intensity, the stickleback control locus amplicon tended to decrease in intensity (Figure 5c). We hypothesize that this may be due to different viral loads in different fish, with the smaller-sized TSIV amplicon outcompeting the larger-sized control amplicon in the pooled PCR reaction when virus DNA is abundant. However, rigorous study of viral loads will require future development of quantitative PCR assays for TSIV. Strong sequencing-based support for varying viral loads comes from the different ratios of viral DNA reads to stickleback DNA reads in the two fish for which genome-wide sequencing data is available. The asterisk lane in Figure 5c corresponds to STMY_X_2011_03, which shows approximately six TSIV copies per diploid stickleback cell based on read depth. The dagger lane in Figure 5c corresponds to STMY_2012_42, which shows approximately 7000 TSIV copies per diploid stickleback cell based on read depth. Despite near 100% TSIV prevalence prior to rotenone treatment, none of the 2024 stickleback (n = 100) showed the strong positivity of all the 2011 and 2012 samples, suggesting 0% TSIV prevalence in stickleback collected in 2024 (Figure 5b, 95% confidence interval: 0 to 3.7%). Thus, the population crash and reappearance of stickleback following rotenone treatment was also associated with the dramatic reduction or loss in apparent TSIV infection in stickleback currently inhabiting Stormy Lake. However, we cannot exclude the presence of low-level infection below the sensitivity of the dual PCR assay.

Based on near 100% TSIV prevalence in 2011 and 2012, the 314 fish translocated to Wik Lake were likely all infected with TSIV and had the potential to spread TSIV to Wik Lake stickleback. However, we found 0% TSIV PCR positivity (n = 100) within Wik Lake in 2024 (Figure 5b, 95% confidence interval for viral prevalence: 0–3.7%). Although it is unknown whether Wik Lake stickleback were ever exposed or infected with TSIV prior to the Stormy Lake fish introduction, exposure to the introduced Stormy Lake fish did not appear to result in a long-term presence of TSIV at high prevalence in the neighboring lake.

## 4. Discussion

In this study, we report the first nearly complete genome sequence of a megalocytivirus infecting a wild North American fish. Although Stormy Lake, Alaska, is located approximately 1893 km from the TSIV-infected stickleback first described by Waltzek et al. [18], the nucleotide sequences from the *MCPs* of the two TSIVs share 99.5% identity, suggesting that they are strains of the same divergent megalocytivirus that Waltzek et al. recommended for a novel species designation. Despite the subsequent characterization of two additional divergent megalocytiviruses (SDDV and ECIV) and recognition of SDDV as a separate species, the taxonomic status of TSIV has not been resolved.

A recent 2018 ICTV proposal (2018.007D) described several criteria for considering viruses members of the same species: similar genomic size, similar GC content, collinear gene arrangement, ≥95% amino acid sequence identity based on concatenated core genes, and phylogenetic relatedness. The 115 kb genome of TSIV is slightly longer than the ISKNV species genomes (110–113 kb) and shorter than the SDDV and ECIV genomes (124–131 kb). With 94 predicted ORFs, the TSIV genome contains fewer ORFs than the other megalocytivirus genomes, which range from 108 to 136 ORFs. The TSIV genome has substantially higher GC content (60%) than any other published megalocytivirus genome, but is more similar to the ISKNV species genomes, which have 53–55% GC content, than SDDV and ECIV, which have 37–39% GC content. The arrangement of genes is most similar to the ISKNV group, but shows two different multi-gene inversions compared to previously described ISKNV genomes. Based on concatenated core gene amino acid sequences, we observe 49.2–70.9% amino acid sequence identity between TSIV and other megalocytiviruses. TSIV also occupies a distinct phylogenetic position as a sister taxon to ISKNV species that is also distinct from SDDV and ECIV. Finally, TSIV is found in a different environment than ISKNV (cool water at northern latitudes rather than temperate and tropical locations). Consequently, TSIV meets multiple criteria established for demarcation as a separate species.

While most of the predicted ORFs share homology with other megalocytiviruses, the TSIV genome appears to have lost and gained key genes. The TSIV genome lacks the transcription elongation factor TFIIS identified as a core gene by Eaton et al. [3]. TFIIS is an evolutionarily conserved protein with roles in the formation and/or stability of RNA polymerase II pre-initiation complexes and transcript elongation by promoting read-through of elongation blocks [53]. Although the absence of this gene could result from an incomplete assembly, the addition of new sequenced iridovirus genomes has shortened the list of conserved core genes, and recent analysis produced a stricter set of 18 core genes that does not include TFIIS [54]. Conversely, the TSIV genome contains three apparently unique ORFs with no homology to other known proteins. These ORFs may represent TSIV-lineage specific innovations.

The TSIV genome also encodes homologs of eukaryotic and other viral proteins, suggestive of more recent lateral gene transfers between host and virus, and between viral groups. Gene acquisition from cellular and viral genomes has been proposed to account for much of the genome expansion of large dsDNA viruses [55,56,57,58,59]. The TSIV genome encodes a homolog of the eukaryotic TRAF2 protein. TRAF2 is involved in signal transduction from TNF receptors, leading to the activation of the NF-kB pathway and JNK pathways, and regulation of apoptosis [60,61]. Though the TSIV TRAF2 shows greater homology to TRAF2 proteins encoded in fish genomes, TRAF2 homologs are also found in ISKNV genomes. ORF111L, the TRAF2 homolog encoded by ISKNV, associated with TRADD and induced caspase 8-mediated apoptosis at higher levels than the cellular homolog of TRAF2 in a zebrafish model [62]. The TSIV TRAF2 may play a similar role in promoting apoptosis in a stickleback host. Modulation of apoptosis by iridoviruses may be beneficial—inhibition of apoptosis during earlier infection stages could promote viral replication, while induction of apoptosis could help the virus disseminate to adjacent cells and limit pro-inflammatory responses [63,64]. The TSIV genome also encodes a homolog of the chordopoxvirus B22 protein, which appears to have undergone at least two but likely three horizontal gene transfer events from poxviruses to herpesviruses and iridoviruses. B22 knockout and gain of function models have shown that a functional B22 protein is not necessary for viral replication in cell culture but is both necessary and sufficient for increased virulence in vivo [50,65,66]. B22 modulates the host immune system by inhibiting antigen presentation to T cells and natural killer cells [50,51,52]. Attenuated poxviruses, such as vaccina virus and certain strains of monkeypox and cowpox, either lack B22 proteins or have mutations inactivating them, making them less virulent [50,65]. The extent to which B22 may impact the virulence of TSIV is unclear, but the repeated sharing of this gene between viral groups suggests it may provide a transferable viral benefit in multiple types of viral infections.

The discovery of TSIV-infected stickleback in Stormy Lake, Alaska, expands the range of TSIV farther north and into freshwater-resident stickleback. Finfish farming is currently illegal in Alaska, so the stickleback in Stormy Lake would likely have limited exposure to other megalocytiviruses previously associated with outbreaks in aquaculture, consistent with the observed divergence of TSIV from other megalocytiviruses. Notably, we did not identify other instances of TSIV in our broader screen of 206 globally distributed freshwater and marine stickleback, suggesting that TSIV infections are not common and widespread in wild stickleback populations. However, previously sequenced genomes are enriched in fish from the Pacific coastal regions of North America and less abundant for Atlantic Ocean and Asian Pacific stickleback, and most wild populations were represented by a single sequenced individual. 

Within Stormy Lake, 100% of fish tested were positive for TSIV in two consecutive years, indicating that stickleback may be able to tolerate persistent iridovirus infections. High prevalence combined with lower virulence of TSIV is consistent with the relatively low mortality rates observed in infected stickleback by Waltzek et al. [18]. Lake-wide rotenone treatment appears to be associated with subsequent loss of TSIV in Stormy Lake. We note that the invasive freshwater plant *Elodea* was also discovered in Stormy Lake during the 2012 rotenone campaign [48], and the lake was subsequently treated with the herbicides diquat and fluoridone in 2014 and 2015 to eliminate this underwater aquatic non-native plant species [67]. Possible mechanisms for loss of the virus include complete death of all infected individuals in the 2012 rotenone campaign, temporary reduction in fish density to a point that transmission could not be sustained, recolonization of the lake by fish more resistant to infection, or limnological changes that prevent sustained transmission. Further experiments could compare genetic markers in pre-rotenone Stormy Lake stickleback, contemporary Stormy Lake stickleback, and surrounding stickleback populations to identify the source of contemporary Stormy Lake fish, which have previously been hypothesized to come from an adjacent outlet stream [48].

Because TSIV no longer appears to be common in Stormy Lake stickleback, future studies of the virus will require identification of other infected populations. Stickleback are already extensively studied in many laboratories [68], and more robust screens for infected fish could be performed using the TSIV genome provided here and additional population genomic studies. If other infected fish are found, attempts to isolate the virus in cell culture could provide opportunities for functional studies of specific TSIV proteins and better characterization of the infection biology. Moreover, if TSIV is detected in multiple additional locations, TSIV isolates could be compared to identify whether any viral sequence differences might correlate with variation in host genome sequences, phenotypes, or environmental parameters. Finally, screening sympatric fish species for TSIV infection could clarify whether TSIV has the capacity to infect other fish hosts, a common feature of other megalocytiviruses.

## Data Availability

The raw sequencing data for the STMY_2012_42 WGS library presented in the study are openly available in the Sequence Read Archive at PRJNA1156815. The two assembled contigs of the TSIV genome are openly available in Genbank at accessions PQ335173 and PQ335174. Code used for analysis is available at github.com/ambenj/TSIV, accessed on 20 October 2024.

## Supplementary Materials

The following supporting information can be downloaded at: https://www.mdpi.com/article/10.3390/v16111663/s1, Table S1: Iridovirus genomes used for annotations, core gene phylogenetic analysis, pairwise identity calculations, and collinearity analysis; Table S2: B22 proteins from poxviruses, iridoviruses, and herpesviruses used to construct B22 phylogeny; Table S3: TSIV genome annotations; Table S4: Pairwise nucleotide identities of the *MCP*; Table S5: Pairwise amino acid identities of 24 concatenated core genes; Figure S1: Mapping of paired reads across TSIV junction positions; Figure S2: Dot plot of the TSIV genome aligned against itself to visualize structural organization and repeats.
