# Supplementary material for: Genomic Sequence of the Threespine Stickleback Iridovirus (TSIV) from Wild Gasterosteus aculeatus in Stormy Lake, Alaska
Source: Viruses. 2024 Oct 24;16(11):1663. doi: 10.3390/v16111663 (PMC11598847; doi:10.3390/v16111663)
Supplement: Supplementary file 1 [file viruses-16-01663-s001.zip › viruses-3255679-figures final.pdf]

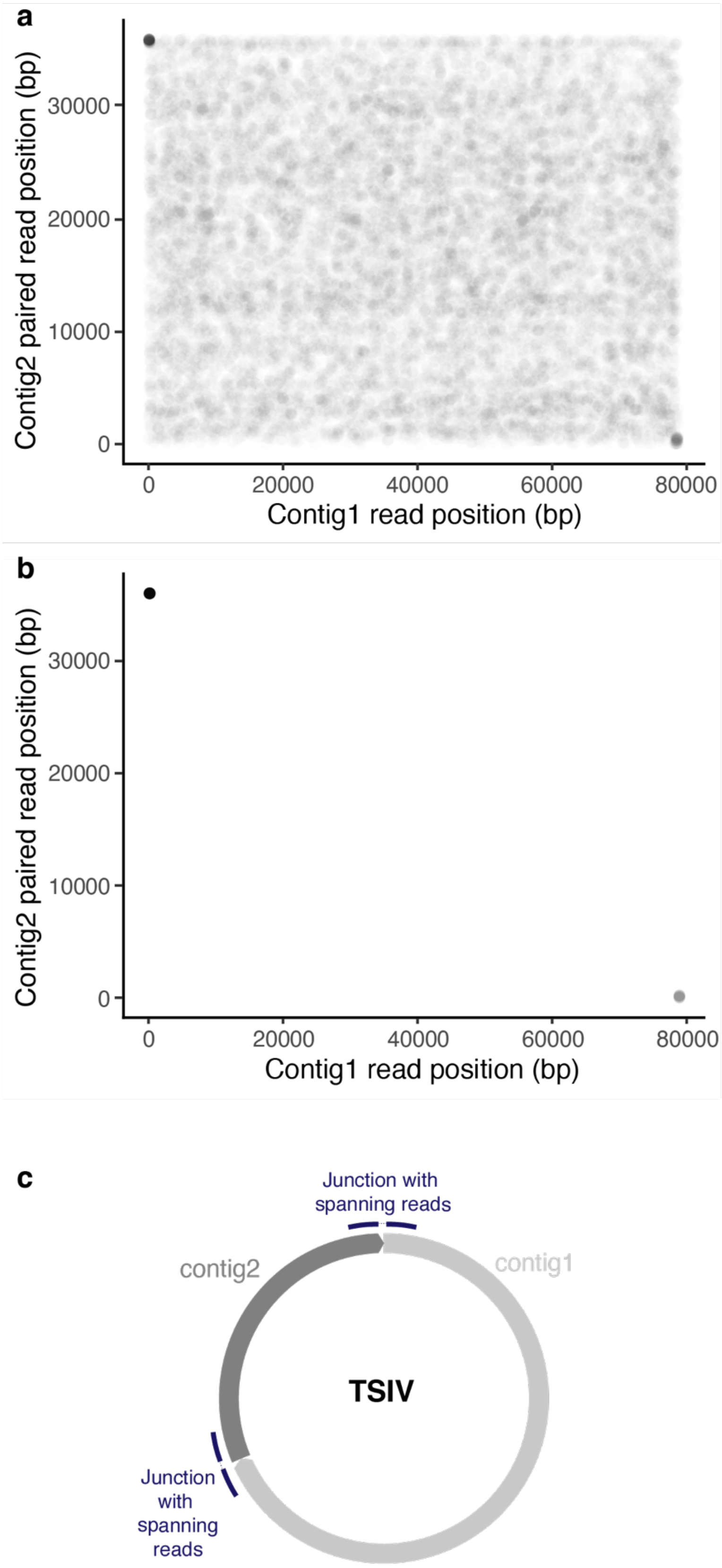

Figure S1. Mapping of paired reads across TSIV junction positions. (**a**) Positions of paired reads from the deeply sequenced stickleback fish (STMY_2012_42) that map to both contig1 and contig2 of TSIV. Darker spots indicate more reads mapped at those positions. (**b**) Similar plot from the paired reads of a less densely sequenced second stickleback (STMY_X_2011_03). This previously sequenced fish led to the initial discovery of TSIV presence in Stormy Lake (see results section 3.1). (**c**) Positions where enrichment of spanning reads in both fish suggest TSIV genome contigs should be connected. Gray arrows indicate the orientations of contig1 and contig2 as they are currently oriented in the TSIV genome assembly.

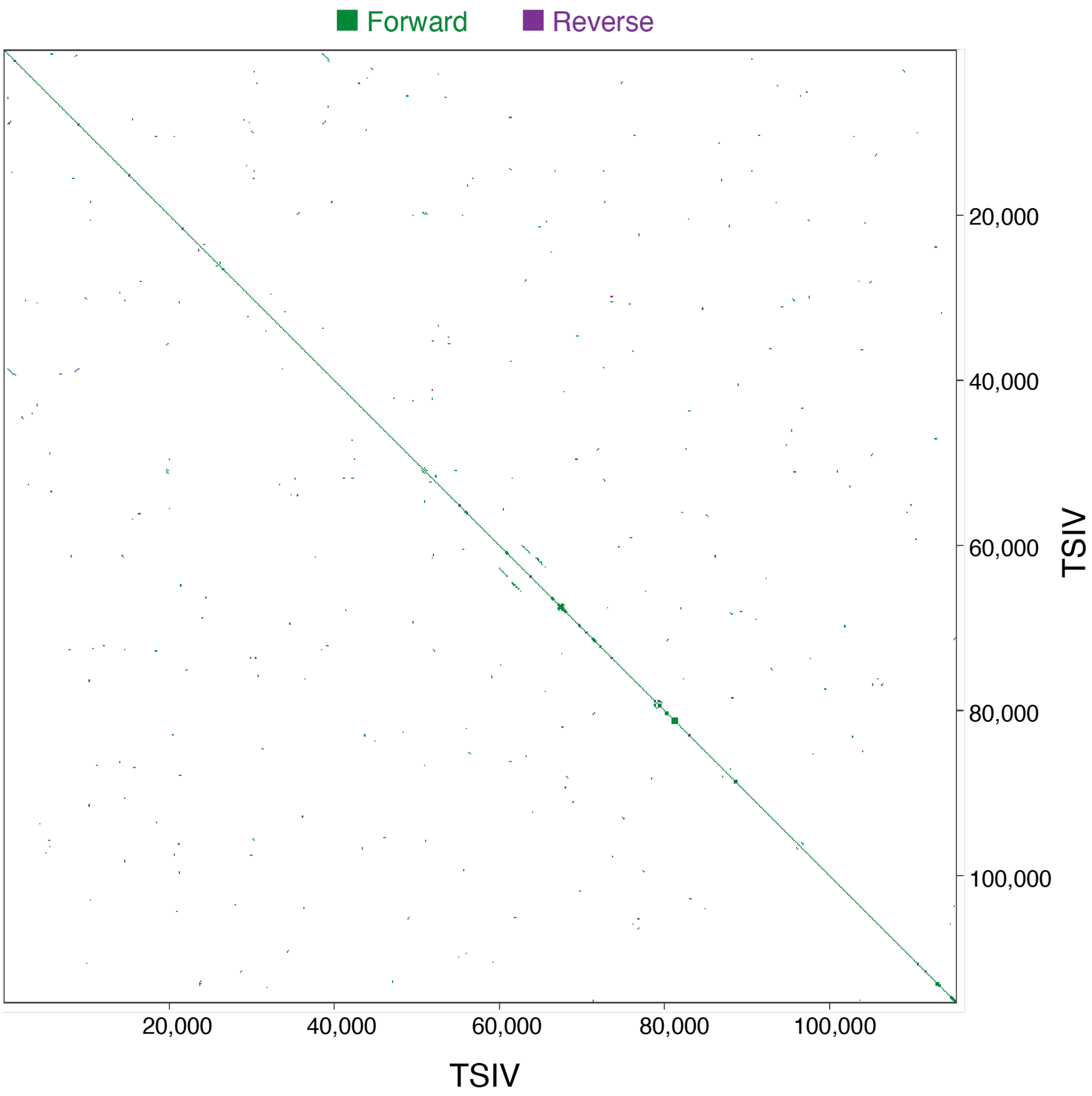

Figure S2. Dot plot of the TSIV genome aligned against itself to visualize structural organization and repeats. TSIV contig1 and contig2 were concatenated with a spacer of $(N)_{100}$ such that contig1 ends at 79,043 bp and contig2 begins at 79,144 bp. Forward and reverse orientation of alignments are indicated by green and purple, respectively.
